# Nighttime lights as a proxy for conflict intensity and infrastructure recovery in Yemen and Ukraine

**DOI:** 10.21203/rs.3.rs-8725596/v1

**Published:** 2026-02-06

**Authors:** Maia C. Tarnas, Tetyana I. Vasylyeva, Volodymyr M. Minin, Daniel M. Parker

**Affiliations:** University of California, Irvine; University of California, Irvine; University of California, Irvine; University of California, Irvine

**Keywords:** nighttime lights, Earth observation, epidemiology, conflict, remote sensing, conflict epidemiology

## Abstract

**Introduction:**

Quantifying the impacts of armed conflict on civilians and infrastructure remains a major challenge, particularly where reporting is limited. Most conflict measurement tools require affected populations to report events and are limited by short time series, under-reporting, and varying methods. These tools do not capture infrastructural rebuilding, which has important health implications. Given this, we demonstrate the utility of nighttime lights (NTL) as a complementary tool for measuring conflict dynamics and infrastructure recovery with an epidemiological application.

**Methods:**

We used monthly NASA Black Marble data to analyze NTL patterns in Yemen (2012–2022) and Ukraine (2019–2024) before and after the onset of large-scale military operations. We calculated month-specific NTL ratios relative to pre-conflict baselines and assessed the alignment of structural breakpoints, identified using BFAST methods, with aerial attack onset. Generalized additive models were used to measure the relationship between NTL and aerial attacks while accounting for the built environment, population, diesel price (Yemen), and spatiotemporal factors. Finally, we applied NTL to an existing model on the association between conflict, measured via air raids, and cholera in Yemen by replacing the original conflict categories with ones defined by NTL and included a variable for NTL recovery.

**Results:**

Mean NTL declined by 53.3% in Yemen and 21.0% in Ukraine following conflict escalation, with detected breakpoints aligning with aerial attack onset in 85.7% of Yemeni governorates and 51.9% of Ukrainian oblasts. Generalized additive models showed that attacks were significantly associated with NTL reductions, independent of built environment factors. Incorporating NTL-based conflict measures into a cholera transmission model for Yemen produced results consistent with attack-based models and found that light recovery was associated with reduced disease risk.

**Discussion:**

NTL is a viable tool for measuring conflict and can offer insights on dynamics that are not present in standard tools while avoiding many of these tools’ limitations. They have epidemiological applications and can be a proxy for important events affecting transmission dynamics. While event-based tools have vast utility, NTL can complement them with specific strengths and means of application.

## Introduction

Armed conflict has profound impacts on people and their environments. Critical civil infrastructure (e.g., roads and hospitals) is frequently damaged or destroyed, contributing to and compounding other adverse conflict effects. Such conditions are primed for infectious disease spread and large-scale population displacement (1, 2). Although these pervasive impacts are well recognized, effects of armed conflict on civilian populations are difficult to measure systematically and quantitatively. Several tools have been developed to monitor armed conflicts using various methodologies. The Armed Conflict Location and Event Database (ACLED) (3) and the Uppsala Conflict Data Program (UCDP) (4), for example, monitor conflict globally and report on individual conflict events. More targeted tools, such as the Yemen Data Project (YDP) (5), monitor specific conflicts, while others, like the WHO’s Surveillance System for Attacks on Health Care, focus on particular types of conflict-related violence. Each of these tools is valuable and has important implications for advocacy and accountability.

Despite their importance, existing tools face limitations. Many rely on secondary reports (e.g., national media outlets), which introduces challenges, particularly in heavily affected areas where communication infrastructure is degraded or where other security or political concerns can prevent accurate documentation of conflict events. As a result, conflict events may be underreported, particularly in regions experiencing the most severe impacts. Delays in documentation, especially in early stages, can also limit the available data. Differences in reporting standards across contexts further complicate comparisons. Importantly, existing tools only capture violent events and actions that cause damage, offering limited insight into broader impacts of armed conflict, such as disruptions to infrastructure and economic activity, as well as recovery. As armed conflicts are often protracted, research arguably should also consider how and where recovery occurs, especially given the role of infrastructure in moderating some health outcomes (6). For example, infrastructure damage can lead to poor sanitation, which can in turn increase the prevalence of diseases associated with poor sanitation; conversely, recovery of this infrastructure can limit the conditions in which these diseases thrive.

Given these gaps, complementary data sources are valuable for reducing potential biases, providing extended time series, and offering recovery insights. Earth observation, particularly nighttime lights (NTL), offers useful tools. Visible and near-infrared light are captured through the Suomi National Polar-Orbiting Partnerships Visible Infrared Imaging Radiometer Suite’s (SNPP VIIRS) Day-Night Band (DNB) sensor at a 15 arc-second spatial resolution. NTL has been applied in several research areas, including light pollution (7–9), human settlement and development (10–12), economics (13), and migration (14). In conflict settings, changes in NTL have been studied descriptively (15) and in the context of power supply disruptions (16, 17), declines in economic activity (18), and population movement and behavior, among other topics (18–20). NTL has also been used by humanitarian and media organizations to track ongoing conflict (21). However, none to our knowledge have quantified the relationship between NTL and conflict events or showed NTL’s utility in infectious disease epidemiology.

We aim to assess NTL as a tool for quantifying armed conflict and recovery during conflict, as well as their impact on health outcomes, in Yemen (**Figure S1**) and Ukraine (**Figure S2**). Yemen has faced protracted armed conflict since late 2014, fought largely between Houthis and the internationally recognized government backed by a Saudi-led coalition (22). The ongoing fighting has left 4.5 million people internally displaced (many of whom have been displaced multiple times) and 66% of the population in need of humanitarian aid and protection (23, 24). The conflict has contributed to widespread infrastructural damage and destruction, economic collapse, and increased disease incidence (1). Recent armed conflict in Ukraine began around a similar time in 2014 with Russia’s annexation of Crimea and its occupation of parts of the Donbas region in the country’s east (25). Following ongoing tension, Russia launched a full-scale invasion of Ukraine on February 24, 2022. As of February 2025, 6.9 million people have fled Ukraine, with an additional 3.7 million displaced internally; close to 35% of Ukraine’s population are in need of humanitarian assistance (26). Russian attacks have caused extensive infrastructural damage, including to water, sanitation, and hygiene (WASH), healthcare, education, and energy sectors.

Using these contexts, we assess the relationship between NTL and reported aerial attacks, in addition to the use of NTL in an existing disease model. We hypothesize that NTL declines largely reflect conflict-related infrastructural damage and destruction; this infrastructure damage has been examined in other remote sensing-based work (15, 27). To be clear, we are not advocating for the use of NTL as a universal conflict detection tool; rather, we aim to demonstrate its use as a complementary and accessible proxy that responds to some of the limitations of event-based reporting and which can offer additional insights with a relatively low barrier to entry. NTL provides another practical tool in our toolbox for understanding conflict dynamics and their association with health outcomes, which is increasingly necessary as conflicts reach heightened levels of violence and protraction globally.

## Methods

In this retrospective analysis, we use monthly Black Marble NTL data (VNP46A3) during January 2012–March 2022 in Yemen and January 2019–June 2024 in Ukraine, extracted using *blackmarbleR* (28). The creation and processing of these products have been described in detail elsewhere (29). Briefly, Black Marble uses the SNPP VIIRS DNB sensor and corrects for atmospheric, thermal, stray light, terrain, and lunar bidirectional reflectance distribution (BRDF) function effects to reduce background noise (30). The monthly products, available beginning January 2012, are composites of daily corrected images with outliers removed; monthly mean values are calculated using remaining observations with contaminated pixels gap-filled and background noise removed (30). In both countries, we utilized the all-angles snow-free observations (see **Figure S3** for view-angle comparison and **Figure S4** for quality and completeness). We also applied a mask at 300 nW·cm^−2^·sr^−1^ in Yemen to remove gas flares from the country’s oil production (15).

Following this processing, we first calculated monthly mean NTL levels for all administrative units and months in the study period. In Ukraine, mean values for months of missing or contaminated data were interpolated by seasonally decomposing the time series, linearly interpolating the adjusted data, and reinserting the seasonal component using the *forecast* package (31). This missingness (occurring in summer months) was expected because of Ukraine’s latitude and Black Marble’s stray light correction methodology. We then calculated month-specific baseline NTL levels by taking the mean light level for each respective month (January–December) across three years of data leading up to the onset of large-scale military operations (our ‘event’) across both countries for a total of 38 months in Yemen and 37 in Ukraine. These baselines were used to calculate the ratio of pre- to post-event NTL levels for each month, referred to as the NTL ratio. Lastly, light recovery was calculated by identifying the post-event two-month rolling NTL minimum (nadir) for each administrative unit and calculating the ratio of light levels for subsequent months. A two-month minimum was chosen to capture nadirs before recovery while limiting the potential seasonal bias seen with shorter periods. Across all analyses, Socotra (Yemen) was not included given its distance from mainland Yemen, and we refer to all of Ukraine’s 27 regions in the first administrative level as ‘oblast’ for simplicity.

To account for the number of reported aerial attacks in each country, we used YDP (5) and ACLED (3). YDP reports on Saudi-led air raids in Yemen between their onset in March 2015 through March 2022. Each reported air raid contains a minimum value of one or more verified air strikes and a maximum value of air strikes, some of which may not be verified. We used the minimum verified number in our study as a conservative measurement of conflict. ACLED likewise provides disaggregated information on conflict events gathered from media sources, reports, and local partners that are cross-referenced and verified. We used reported attacks in the following categories to account for aerial attacks in Ukraine beginning February 2022: shelling/artillery/missile attack, air/drone strike, grenade, remote explosive, and suicide bomb. For both countries, aerial attacks were aggregated monthly for each administrative unit.

We also extracted population size estimates from WorldPop and data on the built environment for each administrative unit. WorldPop provides annual unconstrained UN-adjusted population estimates at 100m through 2020 (32). Years after 2020 were linearly extrapolated. We ran secondary models with population size as the response variable (**Figure S5** and **Table S1**) and sensitivity analyses for all models using Gridded Population of the World data (**Tables S2–S4** and **Figures S6** and **S7**). To account for the amount of built environment in each administrative unit, we used Sentinel-2’s Dynamic World Land Use/Land Cover (LULC) annual data, available at a 10m scale, and calculated the percentage of pixels classified as built from the total number of pixels in each unit (33). In Ukraine, we only used LULC data from summer months to avoid snow cover. Population size and built percentage for both countries were log-transformed to account for skewness. Lastly, we obtained data from the World Food Program on the monthly price of diesel in Yemen, as its electrical grid operates on diesel (34).

### Assessing the alignment of breakpoints and aerial attack onset

We used the Breaks for Additive Season and Trend (BFAST) change detection approach to identify negative breakpoints in the mean NTL time series for each administrative unit while accounting for seasonality (35); this was used to assess whether the onset of attacks was evident in units’ NTL signals, especially given that onset timing varied with countries. BFAST decomposes time series into seasonal, trend, and noise components while estimating the number, timing, magnitude, and direction of abrupt changes within them. If the onset of aerial attacks in the administrative unit fell within the 95% confidence window for a negative breakpoint, we considered the NTL signal as having captured the onset of attacks.

To validate the use of BFAST in this context and better understand the nuances in the time series’ signals, we simulated NTL time series with breakpoints using parameters informed by Yemen and Ukraine’s real data (**Figures S8–S12**). To set the simulation model parameters, we decomposed each administrative unit’s time series into seasonal, trend, and noise components. For each administrative unit, mean trend values were calculated for three periods: pre-break (month 0 to the month prior to the breakpoint), peri-break (12 months following the breakpoint), and post-break (remaining months). In these calculations, breakpoint timing was standardized as month 38 (February 2022) in all oblasts and month 39 (March 2015) in all governorates to align with the onset of armed conflict in both countries. Standard deviation of the noise was also found for each period, adjusted for any short-term pulse in noise around the break. The mean monthly seasonal values were also calculated from the decomposed data for each administrative unit. Using these parameters, median and quartile values across all administrative units in each country were calculated and used to inform a range of parameters for the simulations (**Table S5**). The simulated NTL time series were generated by reconstructing time series from individually simulated trend, noise, and seasonal components.

First, trend values for the pre-, peri-, and post-break periods were sampled from a range of parameters and temporally aligned around the simulated breakpoint. Secondly, noise was simulated using a normal distribution *N(μ = 0, σ = x)*, where *x* was informed by parameters estimated from the real data. Pulses of varying strength (i.e., median, mean, and quartile values) were added to the noise surrounding the breakpoint (4 months prior to the break, month of the break, and 3 months following), though median pulse values were used in presented outputs as there was minimal change in results across the varying strengths. Thirdly, seasonality was set using the median of all administrative units’ mean seasonal values in each country. Lastly, each simulation was given a breakpoint between months 9 and 57 in the Ukraine-informed simulations and 18 and 105 in the Yemen-informed simulations, which account for BFAST’s default minimum segment size of 15% of the time series length. Ten simulations were generated for each set of parameters across every breakpoint (**Figures S9–12**).

### Model building

In both nations we used a generalized additive model (GAM) with a Gaussian response distribution to measure the relationship between NTL (run with both monthly mean NTL and NTL ratio specifications as the response) and reported aerial attacks, while accounting for other potentially important covariates. Both specifications were log-transformed to handle skewness and based on model residuals (**Figures S13** and **S14**). To do so, a small constant was added to all values. In Yemen, we assumed that log-NTL was a linear function of aerial attacks and diesel price, whereas population size and built environment were modeled as splines. To preserve the nonlinear nature of aerial attacks in Ukraine while increasing interpretability, we specified aerial attacks categorically by quintiles using levels of none, low (1–2 attacks), medium (3–6), high (7–117), and severe (118+). Population size and built environment had high concurvity in Ukraine, and built environment was included based on our hypothesis of infrastructural damage. All models included a spline-based function with the study month number as its argument and an interaction term between each administrative unit centroid’s latitude and longitude to account for any baseline temporal and spatial factors that were not captured elsewhere in the model. All GAMs were run with the *mgcv* package in R (36), and equations are in the **Supplementary Text**.

#### Epidemiological application: Cholera outbreak in Yemen, 2016–2019

Mean NTL and light recovery were applied to an existing model on the association between armed conflict and cholera in Yemen during the 2016–2019 outbreak. Raymah was not included in this application as its light recovery ratio could not be calculated given its nadir of zero. The details of the original study, which included data from 20 governorates, can be found elsewhere (1). Briefly, we used case counts from the WHO Eastern Mediterranean Regional Office’s epidemiological bulletins, which report data from the Yemen Ministry of Health and WHO’s joint electronic Disease Early Warning System (37). The model also accounts for population density, vegetation levels, surface water, precipitation, ambient temperature, and economic factors (as a principal component for petrol prices, food basket prices, and the Yemeni rial parallel market exchange rate) using a GAM with a negative binomial distribution.

In the original model, conflict was measured using air raid counts from YDP. The number of verified weekly air raids in each governorate was used to create rolling 3-month aggregate measures which were categorized into severity levels of low (zero air raids in prior 3 months), intermediate (1–4), medium (5–18), high (19–75), and severe (≥ 76). In this application, we used mean NTL to create conflict categories that aligned with the original categories. These were low (0.087–5.904 nW·cm^−2^·sr^−1^), intermediate (0.031–0.086), medium (0.016–0.030), high (0.007–0.015), and severe (0–0.006). The light recovery ratio was also included in the model as an interaction term with the number of months since the light nadir; this was done to attempt to capture related infrastructural restoration while accounting for differences in the speed of recovery. Models were also run using the NTL ratios to define conflict categories, and these can be found in **Table S6** and **Figures S15** and **S16**.

## Results

Aerial attacks and NTL varied spatiotemporally in each country, reflecting the duration of conflict and geopolitical priorities. YDP recorded 25,054 attacks in Yemen between March 2015 and March 2022, primarily in Sa’ada (n = 5,576; 22.3% of total air raids), Marib (n = 3,384; 13.5%), Taizz (n = 2,722; 10.9%), Hajjah (n = 2,587; 10.5%), and Sana’a (n = 2,623; 10.5%) (**Figure S1**). Sana’a City, Aden, and Sa’ada experienced the most air raids per square kilometer at 4.0, 0.50, and 0.49, respectively. Sana’a City’s attack density likely reflects its small size (385km^2^), high population density (mean of 7,806.7 per km^2^), and military importance. Between February 2022 and June 2024, Ukraine experienced 80,608 recorded aerial attacks. Most occurred in Donetsk (n = 30,122; 37.4%), Kharkiv (n = 13,633; 16.9%), Zaporizhzhya (n = 10,258; 12.7%), Kherson (n = 8,308; 10.3%), and Sumy (n = 6,473; 8.0%) (**Figure S2**). These oblasts also had the highest attack density at 1.13, 0.43, 0.38, 0.33, and 0.27 respectively. Note that throughout our analyses, we assessed aerial attacks over the entire oblast, which includes areas under varying political control; this likely influences the timing and distribution of attacks.

**Figure 1:**
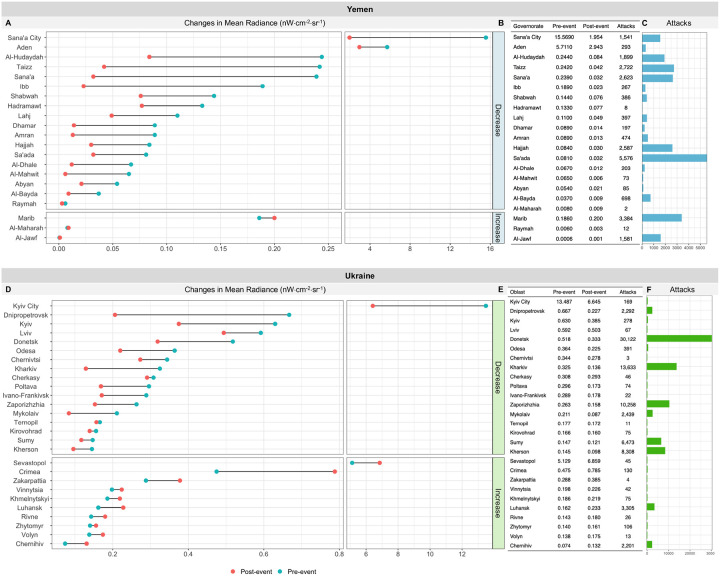
Changes in mean NTL before and after the onset of large-scale military operations. These are defined as March 2015 in Yemen and February 2022 in Ukraine (the ‘event’). Panel A shows radiance changes for Yemen. For example, Sana’a City experienced a decrease in mean radiance from approximately 15.6 nW×cm^−2^×sr^−1^ (blue point) in the pre-event period to 2 nW×cm^−2^×sr^−1^ (red point) in the post-event period. Estimate values for pre- and post-event in Yemen are indicated in the table in B, along with the total number reported aerial attacks (which are also plotted in C). Ukraine’s are presented in panels D, E, and F, respectively. Note the different x-axes for each country.

For the whole of Yemen, NTL generally declined ($\bar{\text{x}}$=−53.3%) following the onset of large-scale military operations, though some areas experienced growth (range: −91.0% to 98.1%). The whole of Ukraine experienced similar effects ($\bar{\text{x}}$=−21.0%, range: −68.2% to 81.1%) (Figs. 1 and S17). In Yemen, NTL in 18 governorates (85.7%) declined; this was greatest in Al-Mahwit (−91.0%), Ibb (−87.9%), Sana’a City (−87.5%), and Sana’a (−86.8%), all in the west. In Ukraine, 17 oblasts (63.0%) had decreased NTL, notably in Dnipropetrovsk (−68.2%) and Kharkiv (−60.1%) in the east, Mykolaiv (−58.8%) in the south, and Kyiv City (−52.3%) in the north. Areas primarily under Russian control experienced some of the greatest NTL increases post-event; this included Crimea (66.6%), Luhansk (41.8%), and Sevastopol (33.5%).

In analyzing light recovery, NTL in 10 governorates (47.6%) reached their NTL nadir between December 2015 and January 2016 (n = 4) or January and February 2016 (n = 6), 9 to 11 months after the onset of air raids. On average, governorates’ mean NTL increased by 1,473.6% from their nadirs; however, NTL levels in the last 6 months of the study period were only 30.9% of pre-event levels. In Ukraine, 11 oblasts (40.7%) reached NTL nadirs beginning 2 months after event onset (March–April 2022), and 7 oblasts (25.9%) had nadirs beginning 7 months after onset (September–October 2022); oblasts subsequently recovered by a mean of 730.4%. Across all oblasts, mean NTL levels in the last 6 months of the study period had recovered to 81.1% of pre-event levels. In both countries, recovery varied heterogeneously across administrative units.

### Alignment of breakpoints and aerial attack onset

**Figure 2:**
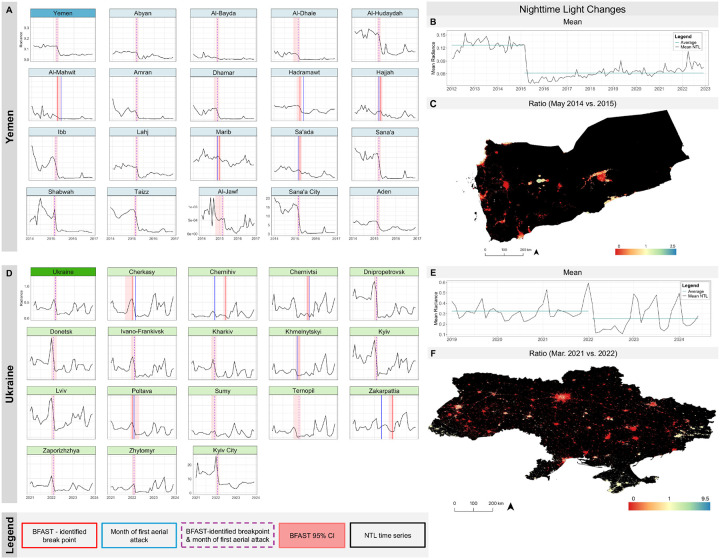
BFAST-identified breakpoints in NTL time series and onset of aerial attacks. The time series (A and D) for each administrative unit is truncated for interpretability. Administrative units for which the NTL signal did not have a detectible negative breakpoint are not shown (Al-Maharah and Raymah in Yemen; Crimea, Kherson, Kirovograd, Luhansk, Mykolayiv, Odesa, Rivne, Sevastopol, Vinnytsya, and Volyn in Ukraine). The full time series for each country is shown in B and E. The maps (C and F) display the overall ratio of mean NTL between the pre- and post-event periods.

The 95% confidence intervals of BFAST-identified negative breakpoints aligned with attack onset in 85.7% (n = 18) of governorates and 51.9% (n = 14) of oblasts (Fig. 2 and **Tables S7 and S8**). The three governorates where these did not align were those with the fewest attacks. In Kherson, Mykolayiv, Odesa, and Vinnytsya oblasts, NTL levels had already been steadily declining over the study period, and the onset of attacks did not meaningfully alter this trajectory. Conversely, Crimea, Luhansk, Sevastopol, Rivne, and Volyn experienced increasing trends that were not notably affected by the full-scale invasion. Kirovograd had no noted change in mean NTL across the entire study period.

In the validation simulations, BFAST successfully identified the true breakpoint in 92% of simulations informed by Yemen’s parameters and 56% of those informed by Ukraine’s. In Yemen-based simulations, accurate identification was primarily driven by a sharp drop in trend values following the breakpoint; seasonality and noise played minimal roles, consistent with their low levels in the real data. In contrast, the Ukraine-informed simulations were noisier and more variable. In these simulations, BFAST was more likely to identify a breakpoint when the trend dropped significantly from the pre- to peri-break period and when a short-term pulse in the noise occurred near the breakpoint—enhancing the detectability of change amid generally noisy conditions. These findings both validate the use of BFAST on the real data and highlight important contextual differences. The mean NTL time series in Yemen had clear signals and limited recovery, which made identifying the signal changes occurring concurrently with attack onset more straightforward. In Ukraine, however, the peri-break period had to be isolated to overcome the masking effects from infrastructure resilience that limited breakpoint identification. Overall, these results suggest that the ability to see the onset of aerial attacks in NTL time series through BFAST or other change detection methods is likely sensitive to underlying country-specific infrastructure resilience and patterns in trend, noise, and recovery.

### Associations between nighttime light and aerial attacks

In Yemen, 14 attacks (the mean number of attacks per month) were associated with a −8.97% (95% CI: −13.40%, −4.31%) change in mean NTL and a −12.38% (−17.94%, −6.45%) change in the NTL ratio (Table 1). All attack quintiles were associated with mean NTL decreases in Ukraine, with the highest indicating a −69.19% (−77.65%, −57.53%) change. Changes in the NTL ratio followed a similar pattern, with a maximum decrease of −59.72% (−69.49%, −46.82%) (Table 1). Spline variable outputs are in **Figure S18**.

### Epidemiological application

The expanded cholera model, which included an interaction term for light recovery, produced similar results across the original conflict specification (air raids) and the NTL-based specification (Table 2 and **Figure S19**).

**Figure 3:**
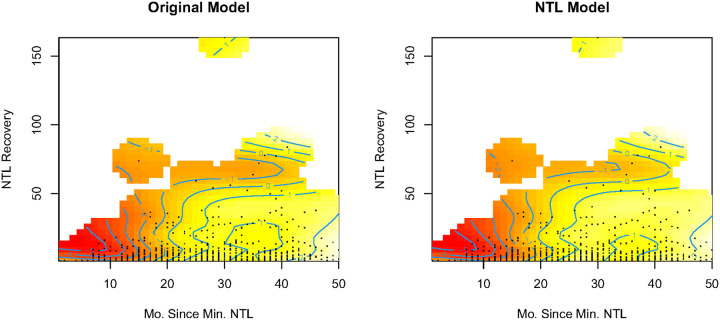
Outputs for the interaction term between light recovery and the number of months since the NTL nadir. Reds indicate lower cholera risk and yellows indicate higher cholera risk after controlling for other variables in the model.

Across all levels of conflict severity, the outcome remained significantly associated with cholera incidence at comparable levels, regardless of which data source was used to specify the conflict categories. The inclusion of the light recovery interaction—which we were unable to do when using just event-based conflict tools—improved model fit (via AIC and deviance explained) across both specifications. Areas that took over 20 months to recover faced increased cholera risk, with the highest risk in areas that had the slowest (> 40 to 45 months) recoveries (Fig. 3). Conversely, areas that recovered quickly (< 10 months) had the lowest risk.

## Discussion

NTL is a viable tool for measuring armed conflict in some conflict-affected settings and is particularly adept at filling in gaps left by event-based conflict measurement tools. This includes providing additional insights on conflict dynamics, such as recovery processes, that are otherwise not normally captured in conflict databases. This work has several practical implications. NTL is not subject to many limitations of event-based tools, including reliability, consistency, lack of standardization, and the impact of conflict on reporting itself. It may also provide a longer time series of available and consistent data, given that the data are temporally constrained only by the satellite’s activation. Use of NTL has a low barrier to entry, especially when compared to advanced tools such as synthetic aperture radar; this makes it more accessible to humanitarian actors during armed conflict. Importantly, NTL does not rely on the affected population to report on or document conflict events (though we advocate for its use to fill gaps in data rather than to replace on-the-ground reports).

In both Yemen and Ukraine, aerial attacks were significantly associated with decreases in NTL at the first administrative unit level. In the majority of instances, the onset of aerial attacks was identifiable in the mean NTL time series through negative breakpoints. This was true in fewer oblasts than governorates, as many oblasts had upward or downward trends that began prior to the full-scale invasion and continued largely unchanged. The course of NTL over the study period in Ukraine also reflected the conflict’s geopolitical context, with areas primarily under Russian control experiencing a growth in NTL following the full-scale invasion. Similar increases were found in other work (18). This may reflect the aggregation of Russian troops in occupied areas and Russian-backed infrastructure development.

The ability of NTL to elucidate regional recovery is a key strength. We hypothesize that much of this light recovery is capturing infrastructure restoration, as infrastructure is a critical component in countries’ physical recovery (38, 39). Ukraine’s recovery resulted in light levels closer to those before the full-scale invasion, whereas levels in Yemen remained low. Yemen’s weak infrastructure pre-conflict and economic devastation have limited its infrastructure restoration, while international aid remains insufficient (40–42). Pre-conflict infrastructure in Ukraine, however, was largely more robust and resilient (43), and international support for recovery has generally been strong (44). Monitoring recovery also has meaningful epidemiological applications. When used in the cholera model, governorates’ cholera risk was modified by their recovery: governorates with fast and strong recovery had the lowest risk, whereas those that had slow and weak recovery had the highest risk. Such findings highlight the importance of infrastructure in disease transmission mechanisms (and the importance of investing in infrastructure recovery during armed conflicts), and NTL gives us a tool to integrate it into epidemiological models.

Using NTL data is not without limitations and caveats, and there are environments in which it may be less useful for our present purposes. In Yemen and Ukraine, population centers are predominately in areas where satellites can capture NTL levels clearly—something that would be difficult in environments where the built environment is covered by forest, for example. Ukraine’s NTL data are impacted by missingness due to its latitude. Black Marble drops pixels heavily contaminated by stray light, meaning that data in northern regions are frequently missing between June and August. We could interpolate data in these months given our time series length, but this does affect the ability to use NTL in northern latitudes. Additionally, NTL changes do not inherently indicate armed conflict (and vice versa – a lack of change or detectable NTL does not necessarily indicate lack of conflict (45)), and NTL should not be used as a universal conflict detection mechanism. Rather, users should establish the presence of armed conflict (or other event which could disrupt NTL, such as a natural hazard or disaster) before using NTL to measure it. Likewise, an area without detectable NTL should not be automatically considered unpopulated (45). NTL data may also be too coarse to detect precise changes, as the pixel size is roughly 450 by 450 meters. This is also true temporally: we used monthly composites given our long study period, and daily images are likely more appropriate in certain instances. However, these may have inconsistent quality and coverage based on cloud cover and other effects. There may be other artifacts (e.g., sensor saturation) that affect NTL quality, though we expect minimal effects on our data given Black Marble’s preprocessing. This study also relies on attack data from YDP and ACLED, which may face reporting biases in areas under frequent attack or that are occupied.

Even in conflict-affected areas, not all changes in NTL may be related to physical changes and could reflect individual behavior or policy. Of particular note in Ukraine is the influence of rolling blackouts following the full-scale invasion that heavily targeted power infrastructure. It is possible that some of the NTL changes we observe in this study reflect these rolling blackouts in the oblasts where they were implemented. While we hypothesize that the overarching NTL changes are due to infrastructural destruction (which also contributes to the decision to implement blackouts), it is difficult to parse out the true cause of NTL changes at this spatial scale, and more research on these mechanisms is needed. However, our data’s monthly temporal scale likely smooths over most of the NTL instability from rolling blackouts, and we observe similar NTL trends in oblasts where blackouts have been used and where they have not.

Issues of spatial and temporal scale are central to the interpretation of NTL. The modifiable areal unit problem, closely related to the ecological fallacy, is a geographic analytic problem that whereby changing the size or configuration of spatial units can alter statistical results (46). A key question, therefore, is the scale at which NTL is both appropriate and analytically informative. We analyze data at the first administrative level because this scale provides a tractable overview of conflict-related change across large areas and time periods and aligns with the spatial resolution of available epidemiological data. However, conflict events and disease transmission occur at finer spatial scales, where spatial autocorrelation is likely and NTL in one unit may reflect events in adjacent areas. Analyses at smaller scales must therefore account for spatial dependence and the relationship between NTL resolution and the geographic extent of the phenomena under study. Areas with no detected light also require contextual interpretation, distinguishing between expected low-light regions and conflict-related disruption. Temporal context is equally important, as NTL observed at a single point in time is difficult to interpret without reference to prior trends or neighboring areas.

When choosing whether and how to use NTL, it is important to identify its use case. In this paper, we are interested in understanding if, at an ecological scale, NTL could serve as a proxy for aerial attacks and capture broad recovery trends. At this scale, NTL can be used by humanitarians to identify affected areas, track large-scale recovery processes (which is also useful for epidemiologists, as illustrated), and visualize conflict impacts, among other purposes. To work at small spatial scales, it is likely that NTL would need to be paired with more granular Earth observation tools in some humanitarian use cases. For example, NTL can direct humanitarian actors where to look, and other tools can provide local granularities and detail. This could include synthetic aperture radar (27, 47) and repeated high-resolution imagery (48). That NTL may not be directly transferable to all spatial scales, however, is not necessarily to its detriment; we advocate that NTL be used complementarily with other Earth observation tools to understand conflict dynamics, and this remains consistent across scales.

## Conclusion

NTL offers a viable tool for measuring armed conflict in some settings and can provide insights on dynamics that are difficult to capture systematically. Its ability to assess recovery has especially strong potential in epidemiological investigations, where the relationship between disease dynamics and infrastructure is crucial in understanding transmission mechanisms. Event-based conflict measurement tools have vast utility in research, advocacy, and accountability, and we show that NTL can complement these tools with specific strengths and means of application. NTL can also be paired with other Earth observation tools, such as synthetic aperture radar, to increase the accuracy and durability of conflict measurements. Continuing to show the effects of armed conflict through a variety of techniques is critical for accountability and to seek relief for civilians.

## Supplementary Files

This is a list of supplementary files associated with this preprint. Click to download.

- SupplementaryInformation.pdf

## Figures and Tables

**Table 1:** Outputs of GAMs showing the association between attacks and NTL (mean and ratio). For attacks, percentage change of the dependent variable was calculated as 100·(β − 1), and each β was calculated by exponentiating the model coefficient. Note that diesel was log-transformed in the model, and thus the β value for a 10% change in diesel price was calculated by raising the base (1.10) to the power of the coefficient. In Yemen, separate GAMs were run with air raids specified individually and scaled ($\bar{\text{x}}$: 14 air raids, SD: 27 air raids).

	Mean NTL		NTL Ratio	
Covariate	\varvec*β* (95% CI)	% change in outcome (95% CI)	\varvec*β* (95% CI)	% change in outcome (95% CI)
**Yemen**				
Air raids				
Individual	0.997 (0.995, 0.998)	−0.35% (−0.53%, −0.16%)	0.995 (0.993, 0.998)	−0.49% (−0.73%, −0.25%)
Scaled	0.910 (0.866, 0.957)	−8.97% (−13.40%, −4.31%)	0.876 (0.821, 0.936)	−12.38% (−17.94%, −6.45%)
Diesel (10% change)	0.976 (0.964, 0.988)	−2.40% (−2.41%, −2.38%)	0.975 (0.959, 0.992)	−2.46% (−2.48%, −2.44%)
**Ukraine**				
Aerial attacks				
None	Comparison		Comparison	
1–2	0.759 (0.649, 0.889)	−24.05% (−35.10%, −11.13%)	0.808 (0.705, 0.927)	−19.18% (−29.54%, −7.30%)
3–6	0.761 (0.631, 0.918)	−23.88% (−36.88%, −8.19%)	0.813 (0.690, 0.958)	−18.69% (−31.00%, −4.18%)
7–117	0.562 (0.438, 0.721)	−43.76% (−56.16%, −27.86%)	0.655 (0.528, 0.814)	−34.47% (−47.23%, −18.62%)
118 +	0.308 (0.224, 0.425)	−69.19% (−77.65%, −57.53%)	0.403 (0.305, 0.532)	−59.72% (−69.49%, −46.82%)

**Table 2:** Outputs of the original cholera model and model using NTL-based conflict categorization. Akaike Information Criteria (AIC) is a model fit statistic where lower values indicate a better fit. Note that the models with recovery do not include Raymah governorate, and the models’ AIC values should therefore not be compared to the AIC of the original model.

	With Recovery		
	Original Model *No. gov. = 20*	Original Model *No. gov. = 19*	Mean NTL Model *No. gov. = 19*
Covariate	IRR (95% CI)	IRR (95% CI)	IRR (95% CI)
Conflict severity			
Low	Comparison	Comparison	Comparison
Intermediate	1.76 (1.51, 2.05)	1.78 (1.52, 2.09)	1.34 (1.06, 1.69)
Medium	1.57 (1.31, 1.89)	1.63 (1.34, 1.98)	1.89 (1.42, 2.52)
High	1.87 (1.53, 2.29)	1.74 (1.41, 2.14)	1.94 (1.38, 2.73)
Severe	2.06 (1.59, 2.69)	2.38 (1.83, 3.09)	1.71 (1.10, 2.64)
Surface water	0.89 (0.78, 1.03)	0.89 (0.78, 1.01)	0.90 (0.79, 1.02)
Precipitation	0.98 (0.91, 1.06)	1.10 (1.02, 1.19)	1.10 (1.02, 1.19)
Vegetation index	0.72 (0.64, 0.80)	0.74 (0.65, 0.83)	0.74 (0.66, 0.84)
Temperature	1.11 (0.91, 1.36)	1.08 (0.90,1 .29)	1.10 (0.92, 1.32)
AIC	30,240.97	28,268.20	28,307.06

## Data Availability

All data are available here: https://github.com/parker-group/NTL_conflict
